# Multi-proxy dentition analyses reveal niche partitioning between sympatric herbivorous dinosaurs

**DOI:** 10.1038/s41598-022-24816-z

**Published:** 2022-12-02

**Authors:** Attila Ősi, Paul M. Barrett, Alistair R. Evans, András Lajos Nagy, Imre Szenti, Ákos Kukovecz, János Magyar, Martin Segesdi, Kinga Gere, Viviána Jó

**Affiliations:** 1 Department of Palaeontology, Institute of Geography and Earth Sciences, ELTE Eötvös Loránd University, Pázmány Péter Sétány 1/C, Budapest, 1117 Hungary; 2 Hungarian Natural History Museum, Baross u. 13, Budapest, 1088 Hungary; 3 Natural History Museum, London, SW7 5BD UK; 4 Monash University, Wellington Rd, Clayton, VIC 3800 Australia; 5 Department of Propulsion Technology HU, Széchenyi István University, Egyetem tér 1, Győr, 9026 Hungary; 6 Department of Applied and Environmental Chemistry, Interdisciplinary Centre of Excellence, University of Szeged, Rerrich Béla tér 1, Szeged, 6720 Hungary; 7 Department of Physical Geography, Institute of Geography and Earth Sciences, ELTE Eötvös Loránd University, Pázmány Péter Sétány 1/C, Budapest, 1117 Hungary

**Keywords:** Palaeontology, Palaeoecology

## Abstract

Dentitions of the sympatric herbivorous dinosaurs *Hungarosaurus* (Ankylosauria, Nodosauridae) and *Mochlodon* (Ornithopoda, Rhabdodontidae) (Santonian, Hungary) were analysed to investigate their dietary ecology, using several complementary methods—orientation patch count, tooth replacement rate, macrowear, tooth wear rate, traditional microwear, and dental microwear texture analysis (DMTA). Tooth formation time is similar in *Hungarosaurus* and *Mochlodon*, and traditional and DMTA microwear features suggest low-browsing habits for both taxa, consistent with their inferred stances and body sizes. However, *Mochlodon* possesses a novel adaptation for increasing dental durability: the dentine on the working side of the crown is double the thickness of that on the balancing side. Moreover, crown morphology, enamel thickness, macrowear orientation, and wear rate differ greatly between the two taxa. Consequently, these sympatric herbivores probably exploited plants of different toughness, implying dietary selectivity and niche partitioning. *Hungarosaurus* is inferred to have eaten softer vegetation, whereas *Mochlodon* likely fed on tougher material. Compared to the much heavier, quadrupedal *Hungarosaurus*, the bipedal *Mochlodon* wore down more than twice as much of its crown volume during the functional life of the tooth. This heavy tooth wear might correlate with more intensive food processing and, in turn, could reflect differences in the metabolic requirements of these animals.

## Introduction

Ornithischian dinosaur feeding traits (tooth morphology, tooth formation, tooth replacement, tooth wear and jaw mechanics) displayed substantial variation during the 140 million year history of the clade (e.g.^1–20^). Ornithopods exhibit some of the most elaborate dentitions and jaw mechanisms^11,21–27^, but it is now clear that thyreophorans also had varied ways of processing food^28–36^. In both groups, complex jaw mechanisms, distinct from the simple orthal jaw closure that was symplesiomorphic for the clade, were present in numerous taxa, and allowed more efficient chewing (and subsequent digestion) of the food consumed. A large body of work has been devoted to reconstructing the evolutionary history of the dental and cranial features associated with feeding in Ornithischia (e.g.,^11,37,38^), but only a few studies have investigated possible differences in the feeding behaviours of sympatric taxa (e.g.^30,39^).

In this paper, we conduct a comparative study of two ornithischian dinosaurs from the Late Cretaceous (Santonian) of Iharkút (Bakony, Hungary, Central Europe)—*Hungarosaurus tormai* and *Mochlodon vorosi*, a nodosaurid ankylosaur and rhabdodontid ornithopod, respectively – which were abundant in this fauna. Here, we aim to determine if these co-occurring, low-browsing herbivores (with maximum browse heights of ~ 1 m above ground-level), exploited the available vegetation in similar or different ways and to use this information to explore their comparative dietary ecology. In order to characterize their feeding habits in as much detail as possible, we compared 15 dental characters (see Table 1) using several different, cross-validating methodologies (comparative morphology, dental histology, traditional microwear, dental microwear texture analysis [DMTA], orientation patch count [OPCR] analysis, 3D modelling and µCT-scanning), representing the first time that all of these methods have been applied in concert to any dinosaur taxon.

**Table 1:** Comparison of feeding-related dental characters in *Hungarosaurus* and *Mochlodon* used in this study.

Variables	*Hungarosaurus*	*Mochlodon*
Tooth count/jaw	21	10
Complete tooth crown volume (mm^3^)	136.28	290.81
Total volume of worn crown per jaw quadrant (mm^3^)	798	1684.3
Mean % of the worn crown/complete crown	28	58
Type of jaw movement	Palinal	Orthal
Max. tooth formation time (days)	126	140
Mean width of von Ebner incremental bands (µm)	16.97	33.55
Min. of estimated tooth replacement rate (days)	120	140
Mean enamel thickness (µm)	20–25	37.2–60
Wear facet angle relative to apicobasal axis (°)	5–45	20–65
% of wear facet/occlusal crown area	20–60	28–93
% of microwear pit/feature number	72.67	82.54
Mean tooth wear complexity (Asfc)	3.393	3.07
Mean tooth wear anisotropy (epLsar)	0.0183	0.0178
Tooth crown complexity	21.75	20.5/20.175

## Material and methods

### Specimens

The specimens used in this study were collected between 2001 and 2021 from the approximately 30 cm thick, Szál-6 bone-bearing bed of the Upper Cretaceous (Santonian) Csehbánya Formation, Iharkút, western Hungary. All specimens are from the same stratigraphic horizon. For reviews of the geology, faunal composition, and taphonomy of this site see^40–42^. All of the jaw elements and teeth were collected as isolated specimens, except for the dentary (MTM 2007.25.2) of *Hungarosaurus tormai*, which is part of the fifth skeleton of this species. All of the specimens are housed in the Vertebrate Paleontological Collection of the Hungarian Natural History Museum, Budapest, Hungary (MTM). See Table 2 for a list of all of the material sampled.

**Table 2:** Details of the specimens used for histological study, and tooth formation times counted on the Hungarian and some related taxa.

Taxon	Inventory number	Tooth position	Complete tooth crown height (mm) * estimated	Tooth formation time (days)	Mean width of von Ebner incremental bands (μm) * lingual/labial	Direction of section
*Hungarosaurus*	PAL 2022.4.1	Maxilla	8.68*	104	19.23	Longitudinal
*Hungarosaurus*	PAL 2022.5.1	Maxilla	7.37	105	16.78	Transverse
*Hungarosaurus*	PAL 2022.6.1	Dentary	7.05	79	17.69	Transverse
*Hungarosaurus*	PAL 2022.7.1	Maxilla	4.99	68	18.01	Transverse
*Hungarosaurus*	PAL 2022.8.1	Maxilla	5.2	63	15.21	Transverse
*Hungarosaurus*	PAL 2022.9.1	??	10.7*	126	18.10	Longitudinal
*Hungarosaurus*	PAL 2022.10.1	Maxilla	9.7*	116 – 125	18.77	Longitudinal
*Edmontonia*^56^	?	?	12*	279	13.9	?
*Pinacosaurus*^34^	IGM 100/3186	?	3.5*	75	15.7	Transverse
Teete stegosaur^36^	ZIN PH 41/246	?	4.0*	95	16.24	Longitudinal
*Mochlodon*	MTM VER 2016.2551. #37L	Maxilla	12.5*	min. 65	49.53	Transverse
*Mochlodon*	MTM PAL 2012.18.1. #35D	Maxilla	11.8*	140	27.57	Transverse
*Mochlodon*	MTM PAL 2016.964._1	Dentary	10.0*	82	31.77	Transverse
*Mochlodon*	MTM PAL 2016.964._2	Maxilla	6.8*	77	31.11/18.21*	Transverse
*Matheronodon*^57^; this study	MMS/VBN-93-34	Maxilla	30*	min. 110	24.55	Horizontal/coronal

## Methods

### Preparation

Each specimen was prepared mechanically in the laboratories of either the Department of Paleontology, Eötvös Loránd University or the Hungarian Natural History Museum. Broken specimens were reassembled using cyanoacrylate glue. Great care was taken to avoid damage to the tooth crowns and their wear facets during collection and preparation, and wear facets were not treated with consolidants or coatings where possible.

### Thin-sectioning

Seven teeth of *Hungarosaurus tormai* and four teeth of *Mochlodon vorosi* were thin-sectioned (Table 2). These thin-sections were prepared following the standard procedure described by^43^. Due to the fragility of the teeth, several additional steps were required: after embedding the tooth crowns in resin (Araldite 2020 epoxy resin) and cutting with a Buehler Isomet 1000, the cut surface was treated with additional resin, sanded with grit abrasives (standard grades 600, 800, and 1000), and then bonded to a glass microscope slide. Subsequently, the surface was treated with resin again, after which it could be sanded down to a final thickness of approximately 70 µm.

### Imaging and measurements

Images of the thin-sections were taken with a QImaging MP5.0 digital microscope camera using a Nikon LV 100 polarized light microscope, and processed with Image Pro Insight v. 8.0. Measurements from the teeth and thin-sections were taken using ImageJ v. 1.48^44^. We redrew the lines between the von Ebner incremental bands (VEIB) revealed in the thin-sections using Photoshop and measured the thicknesses of the tissue between these lines using ImageJ. The use of the term ’von Ebner incremental line’ in the earlier literature is not always clear as to whether the line is a band composed of both its light and dark parts or the boundary between the daily formed bands. Here, we refer to the unit consisting of one dark and one light part as the von Ebner incremental band (VEIB), which we believe is a better indication of its extent.

### 3D modelling

Eleven teeth of *Hungarosaurus tormai* and 25 teeth of *Mochlodon vorosi* were used for 3D modelling. 3D models from the teeth were created using a Polyga HDI Compact S1 3D Scanner. Before scanning, the specimens were coated with either a Pfinder 871 solvent-based aerosol spray or talcum powder in order to reduce the reflections from their shiny surfaces. The specimens were scanned on a turntable connected to the scanner. The point clouds from the multi-directional scans have been precisely merged and the surface generated in FlexScan3D v. 3.3.21.8. The resulting 3D polygon files had polygon lengths between 30 and 80 µm. Further operations were performed in Geomagic Wrap version 2017.0.2.18 (3D Systems, Rock Hill, SC). Measurements of the worn surface area and crown volumetric data were also taken using the latter software (see macrowear and volumetric data in Supplementary data 1).

### Tooth crown complexity analysis (OPCR)

3D models of one unworn *Hungarosaurus tormai* tooth and two unworn teeth of *Mochlodon vorosi* were trimmed to the crown area and oriented in Geomagic Wrap with the occlusal surface pointing in the positive Z direction. Point clouds were exported from Wrap and imported into Surfer Manipulator^45^. Each tooth was standardised to 50 data rows per tooth (RPT) and 25 RPT in mesial-distal direction. Orientation Patch Count Rotated (OPCR) was calculated using eight cardinal directions of 45º, a minimum patch size of three and repeated at eight rotations^46,47^.

### Traditional and DMTA tooth wear analysis

Four *Hungarosaurus tormai* and five *Mochlodon vorosi* teeth were analysed using traditional (2D) microwear and DMTA (3D) analysis. Macrowear is defined herein as a wear feature larger than 0.5 mm, whereas microwear features are considered to be smaller than this. Scratches and pits represent most of the wear features seen on the teeth. Following^48^, pits are defined as having length–width ratios < 4:1. For scratches, this ratio is > 4:1. Alongside the 3D models (see above), light microscopy was used to record gross macrowear patterns. Microwear analysis was performed with a Leica DCM3D confocal microscope (Széchenyi István University, Győr, Hungary). For traditional (2D) microwear analysis four micrographs were saved per tooth as greyscale images using the intensity data from the confocal dataset. Each measured micrograph has a resolution of 768 × 576 pixels, corresponding to a 637 × 477 μm FOV. Measurements were carried out using a Leica HC PL Fluotar EPI 20X lens after a series of sensitivity tests with 20X, 50X and 100X lens. Due to the nature of the investigated samples, no additional information could be gained with higher magnification lens i.e. the increase in level of detail of the 3D dataset with a 100X lens was negligible. On the other hand, using a 20X lens allowed for a quicker acquisition of a comparably larger area with a lateral (X and Y) resolution of 0.83 micron and a vertical resolution of 0.015 micron. It should be noted that the used FOV and spatial resolution is larger than the typical FOV used in DMTA analyses. Nonetheless, 2D analysis based on intensity maps showed a very high level of detail, and therefore should produce comparable results. Images of the microwear facets were analyzed using Microware v. 4.0 following the procedure described by^49^. The generated 768 × 576 pixel grayscale images were viewed on a 27″ Full HD monitor for 2D analysis, which corresponds to a physical image size of approximately 24 × 18 cm (assuming a pixel density of 81 pixel per inch) when viewed at 1:1 scale. The slides were scaled 1:1 in Microware before conducting the 2D analysis. 2D microwear analysis was conducted by the same operator. Four variables were assessed from the micrographs: (1) the percentage incidence of pitting; (2) mean scratch width; (3) mean pit width; and (4) mean pit length. We also report the number of features measured and the standard deviation of means (Supplementary data 2).

Mallon and Anderson^30^ analysed most of the wear facets included in their study over 0.4 × 0.4 mm areas. Consequently, their analysis covered mainly the softer, orthodentine part of the wear facet. By contrast, we analysed four larger areas (0.63 mm × 0.48 mm) per tooth, concentrating on the more resistant mantle dentine located directly under the enamel. In addition,^30^ identified an average of 44.26 features (scratches and pits) across all of the taxa they studied. By contrast, on the four *Hungarosaurus tormai* and five *Mochlodon vorosi* teeth sampled, we identified averages of 281.43 and 404.97 features, respectively. This discrepancy could be due to our inclusion of a high number of small, point-like features on our micrographs that might have not been counted in^30^ analyses, which could account for the much higher number of pits reported in our sample (see “Results” section, below).

3D topographic data from the wear surfaces were collected using the same confocal technique. Raw measurements were post-processed in Mountains v. 8. Datasets were leveled using a least-squares plane method leveling algorithm. Non-measured points were filled with a smooth spline method. No additional data processing was conducted before surface analysis. This approach was chosen to accelerate 3D analysis and to avoid potential misinterpretation of surface features, minimize subjectivity and increase reproductivity. A 500 × 500 pixel area was extracted from each micrograph for evaluation purposes. Each 3D topographic dataset was analyzed by scale-sensitive fractal analysis (SSFA) based on several previous studies (e.g.^50–54^). In the present study the attributes of SSFA measured are anisotropy (epLsar = exact-proportion length-scale anisotropy of relief), complexity (Asfc = area-scale fractal complexity), scale of maximum complexity (Smc), and heterogeneity of area-scale fractal complexity (HAsfc(9 × 9)). The selected parameters are summarized in Supplementary data 2.

For visualization purposes, non-measured points were filled using a smooth shape calculated from the neighbouring points. The resulting surfaces were exported in ‘.sur’ format. A MATLAB algorithm was used to create an automated export of 3D pseudocolor topography maps of the micrographs.

Data of the 2D and DMTA analysis have been statistically analysed with R Statistics Software (version 4.0.5.^55^). For a multivariate analysis, Principal Components Analysis (PCA) has been used (see Supplementary data 2). We used the prcomp() function (within the stats package) with two arguments, "scale" and "center", whose value is TRUE. The summary() function shows the PCA object (the standard deviation, the proportion of variance and the cumulative proportion). The ggbiplot() function (within ggbiplot package) was used for illustration with some arguments ("ellipse", "geom.ind", "pointshape", "pointsize", "obs.scale", "var.scale", "groups").

### Computed tomography

Micro- and nanoCT-imaging was used to investigate the internal structure of the teeth and jaw elements and to reveal replacement teeth. One dentary of *Hungarosaurus tormai* and three dentaries of *Mochlodon vorosi* were CT scanned. MicroCT scanning of the bones was conducted in the laboratory of the Carl Zeiss IMT Austria GmbH (Budaörs, Hungary), using a Zeiss Metrotom computer tomograph with interslice distances of 130 µm. In addition, nanoCT with voxel sizes between 50 and 600 nm was used to detect further details of the jaws and teeth, using a Bruker Skyscan 2211 nano-CT cone-beam scanner (Skyscan, Bruker, Belgium) at the University of Szeged, Hungary. The images acquired were reconstructed in volumetric NRecon Reconstruction (Skyscan, Bruker, Belgium), which allows the correction of typically occurring artifacts (e.g., beam hardening or ring artifacts). For 3D visualization CTVox 3D Micro-CT Volume Rendering (Skyscan, Bruker, Belgium) was used.

## Results

### Tooth formation time

**Figure 1:**
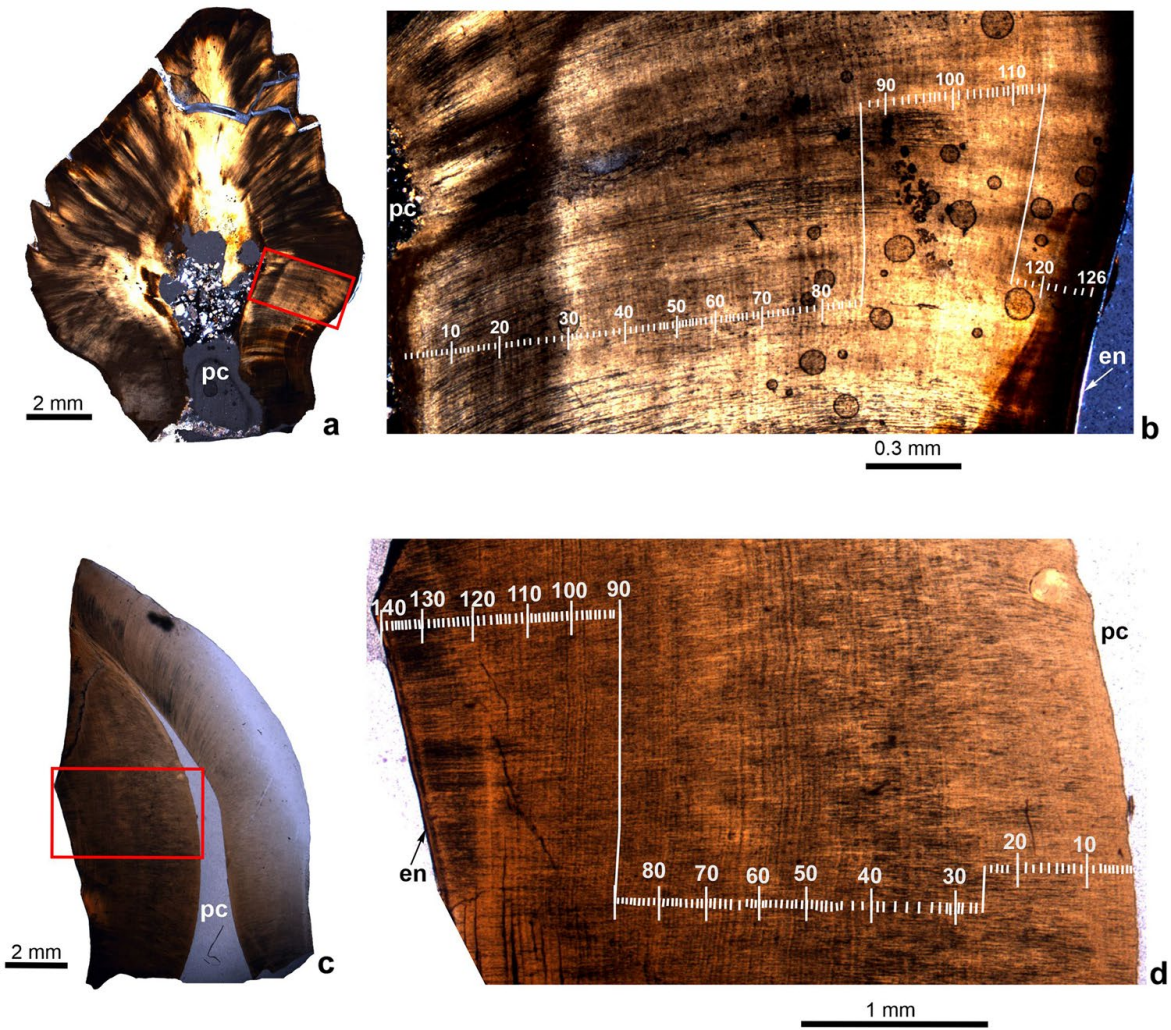
Histological thin-sections of the teeth of *Hungarosaurus tormai* and *Mochlodon vorosi* showing the numbers of von Ebner’s incremental bands (VEIB) counted. (**a**, **b**) *Hungarosaurus tormai* (mesiodistal section). (**c**, **d**) *Mochlodon tormai* (labiolingual section). Abbreviations: en, enamel; pc, pulp cavity.

Tooth formation times are summarized in Table 2. According to^56^, VEIB that can be observed in thin-sections or on the cut, etched surfaces of tooth crowns represent daily tooth formation time. In *Hungarosaurus tormai*, based on four transverse (labiolingual) and three longitudinal (mesiodistal) sections of seven isolated but well preserved specimens, tooth formation time is 63–126 days (Fig. 1a,b) with an average of 94.14 days. This duration is intermediate between those reported for *Pinacosaurus grangeri* (75 days^34^) and *Edmontonia* (279 days^56^). The mean widths of VEIB in the transverse (labiolingual) and longitudinal (mesiodistal) sections are 16.97 µm and 18.7 µm, respectively. These values are higher than in *Edmontonia* (13.9 μm^34^) or *Pinacosaurus grangeri* (15.7 μm^34^). In five of the seven thin-sections, the thickness of VEIB decreases slightly towards the enamel-dentine junction, whereas in the other two taxa the opposite is true. The average enamel thickness in *H. tormai* is 20–25 μm, with slightly thicker enamel around the marginal denticles.

Based on four transverse (labiolingual) thin-sections made from four teeth, the tooth formation time in *Mochlodon vorosi* is 77–140 days (Fig. 1c,d), depending on tooth size (see Table 1). Average tooth formation time is 90.25 days. The mean width of VEIB in the transverse cross-sections is 33.55 µm. In transverse cross-section the crowns of *M. vorosi* are asymmetrical around the pulp cavity (Fig. 1c). This is most clearly observed in the maxillary tooth MTM VER 2016.964_2, where the lingual side of the crown is almost twice as wide as the labial one. In this tooth crown, 61 VEIB are present lingually and 77 labially, but the lingual side is slightly worn so this is a minimum count. The mean thickness of VEIB differs markedly between the lingual (31.11 µm) and labial (18.21 µm) sides of the crown, demonstrating that during tooth formation the amount of dentine formed on a daily basis on the lingual (working) side was almost double that of the labial (balancing) side. The opposite is true in a dentary tooth (MTM VER 2016.964_1), although not to the same extent. In this tooth the labial (i.e. working) side of the crown (measured between the thin pulp cavity and the labial margin of the crown) is approximately 20% thicker than the lingual side. The VEIB on the labial side are similarly thick (31.77 µm) as those on the lingual side of the maxillary teeth. In contrast to the relatively thin enamel of *H. tormai*, the average enamel thickness of *M. vorosi* is 37.2–60 µm (see also^38^). In *Matheronodon provincialis*, based on a coronal section of a tooth^57^, a tooth formation time of 50–100 days has been calculated. However, counting the number of VEIB on Fig. 4e of^57^ results in a minimum tooth formation time of 110 days, and the mean width of VEIB is 24.55 µm.

### Tooth replacement and replacement rate

**Figure 2:**
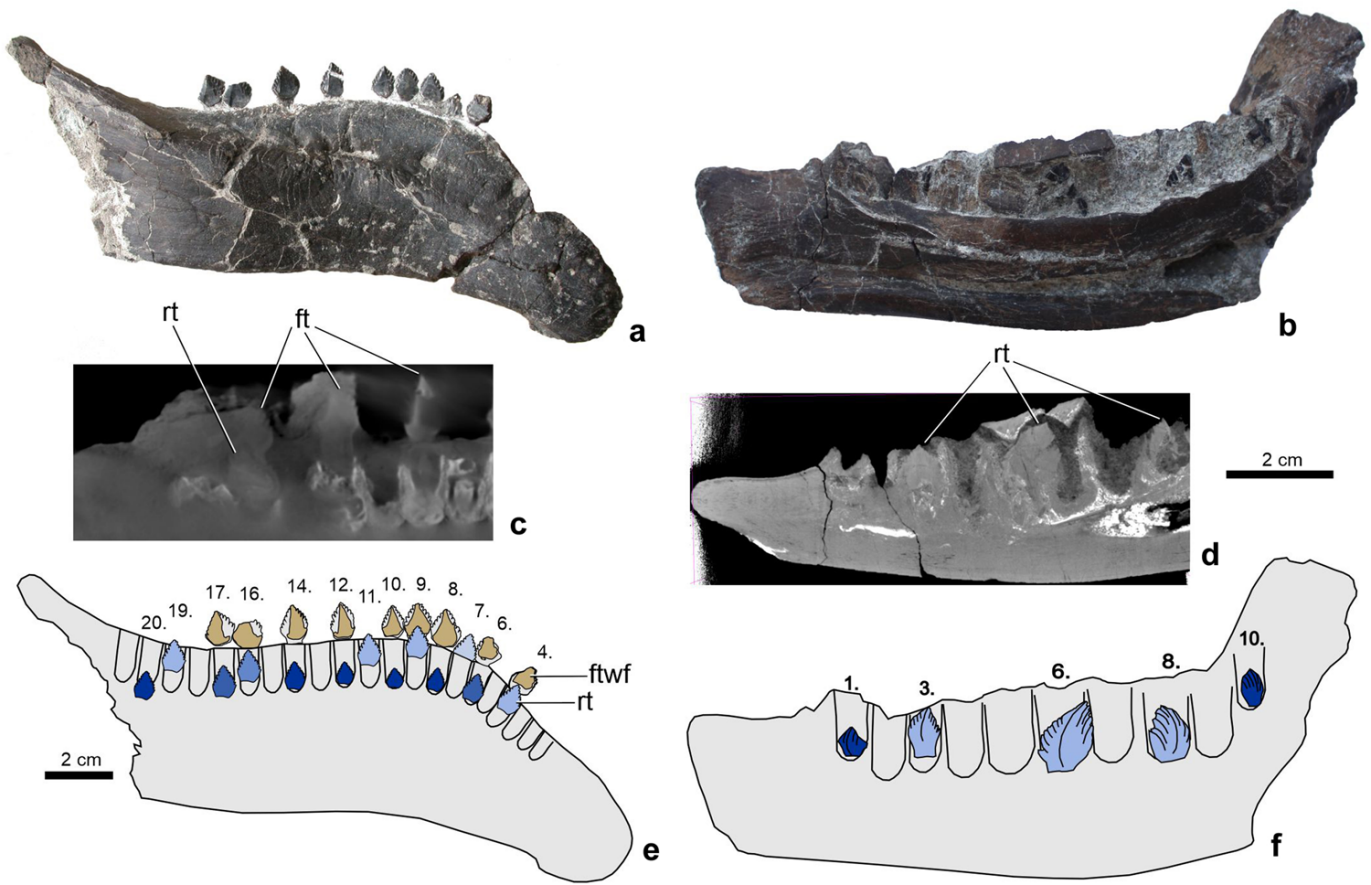
Dentaries of *Hungarosaurus tormai* and *Mochlodon vorosi* that were micro- and nanoCT scanned to reveal replacement teeth. (**a**) Lateral view of the right dentary of *Hungarosaurus tormai* (MTM 2007.25.2) with functional and replacement teeth. (**b**) Medial view of the right dentary of *Mochlodon vorosi* (MTM V 2010.104.1) with replacement teeth. (**c**) microCT scan of the right dentary of *Hungarosaurus tormai* showing replacement teeth. (**d**) nanoCT scan of the right dentary of *Mochlodon vorosi* showing replacement teeth. (**e**, **f**) Schematic drawings of the dentaries of *Hungarosaurus tormai* and *Mochlodon vorosi* showing the functional and replacement teeth. ft, functional tooth; ftwf, wear facet on the functional tooth; rt, replacement tooth.

MicroCT scanning of the dentaries of *Mochlodon vorosi* and *Hungarosaurus tormai* revealed the number, sizes and positions of the replacement teeth. One right dentary of *H. tormai* (MTM 2007.25.2) possesses well-preserved functional teeth in the 4th, 6th–10th, 12th, 14th, 16th, and 17th alveoli (Fig. 2a). Only one generation of replacement teeth is present below these but they are not seen in all alveoli. Replacement teeth are lingual to the root of the functional tooth, as typically observed in most archosaurs^58^. Tooth families (i.e. one functional and one replacement tooth) represent different stages of eruption through 13 alveoli containing at least one tooth generation (total number of alveoli is 21, the remaining eight alveoli are empty; Fig. 2a,e). In the first stage (e.g. 7th alveolus) the partly erupted crown is unworn and no replacement tooth is present. In the second stage, the teeth (e.g. 10th, 12th) were already in use (minimal labial wear present) and a smaller replacement tooth is present, nested deeply beneath the functional tooth. In later phases, as the functional teeth became more heavily worn (e.g. 4th, 16th), the replacement teeth became larger and moved closer to the functional crown (Fig. 2c,e). The pattern of tooth replacement seen in *H. tormai* is similar to that reported in other ankylosaurs^58^; AŐ, pers. obs.).

Functional teeth were lost from all of the preserved *Mochlodon vorosi* dentaries in our sample (Fig. 2b), so the relationships between these and the replacement teeth could not be studied. In two of the three CT-scanned dentaries (MTM 104.1, MTM PAL 2019.93.1), five of the 10 alveoli (1st, 3rd, 6th, 8th, 10th in both specimens; Fig. 2b,f) house replacement teeth (Fig. 2d), whereas in the third specimen (MTM PAL 2019.83.1) only the 3rd, 5th, 7th, and 10th alveoli possess replacement teeth. Suggested ontogenetic size differences are not related to the different replacement tooth count, as PAL 2019.83.1 is almost twice as long (compared by the anteroposterior length of the alveolar row) as PAL 2019.93.1.

Tooth replacement rates could not be calculated using the non-invasive approach of^59^, because no specimens are available for either taxon where both the functional and replacement tooth generations are intact, so differences in their formation times could not be estimated. In *Hungarosaurus tormai*, all of the fully erupted teeth in the most complete dentary are already worn (Fig. 2a), and below them are teeth of the same height as the functional tooth or, at most, one-third smaller. However, the method of^59^ requires a complete functional-replacement tooth pair (and estimations between different replacement generations cannot be made here as *H. tormai* has only one replacement tooth generation). The most complete functional tooth is found in the 7th alveolus of the right dentary (MTM 2007.25.2, Fig. 2a,e), which is unabraded with ~ 60% of the crown erupted. This tooth is nearly identical in size to those with documented formation times of ~ 126 days. However, microCT-scans show that there is no clearly identifiable replacement tooth below, only a tiny (diameter ~ 2 mm) shapeless, incompletely formed tooth. This suggests that the minimum tooth replacement rate for *H. tormai* may have been the same as, or slightly less than, the tooth formation time (minimum of ~ 120 days). For comparison, the minimum tooth replacement rate in *Pinacosaurus grangeri* is 53 days (but was presumably higher, given that this is a juvenile specimen^34^), which is nearly proportional to that of *H. tormai* when scaled for tooth size (the teeth of *H. tormai* are approximately two times larger than that of *Pinacosaurus grangeri* based on mesiodistal width). In *Edmontonia*, whose teeth are at least four times larger by volume than those of *Pinacosaurus grangeri*, and 1.5–2 times larger than those of *H. tormai*, the tooth replacement rate was presumably much longer than in *H. tormai* based on the small size of replacement teeth of *Edmontonia*^34^.

The situation may have been similar in *Mochlodon vorosi*, although there are no functional teeth in the sampled jaws (Fig. 2b). There is only one tooth in MTM 2019.83.1, one-third of which is erupted, unabraded and that is the same size as the largest tooth examined histologically. However, there is no sign of a replacement tooth below this, suggesting that the tooth replacement rate in this taxon was also relatively high, and at least equal to the time taken for tooth formation (i.e. minimum of 140 days). By comparison, in the few other ornithopods in which tooth replacement rates are known these rates are generally higher (e.g. 50–80 days in some hadrosaurids^56^).

### Macrowear and wear rate

**Figure 3:**
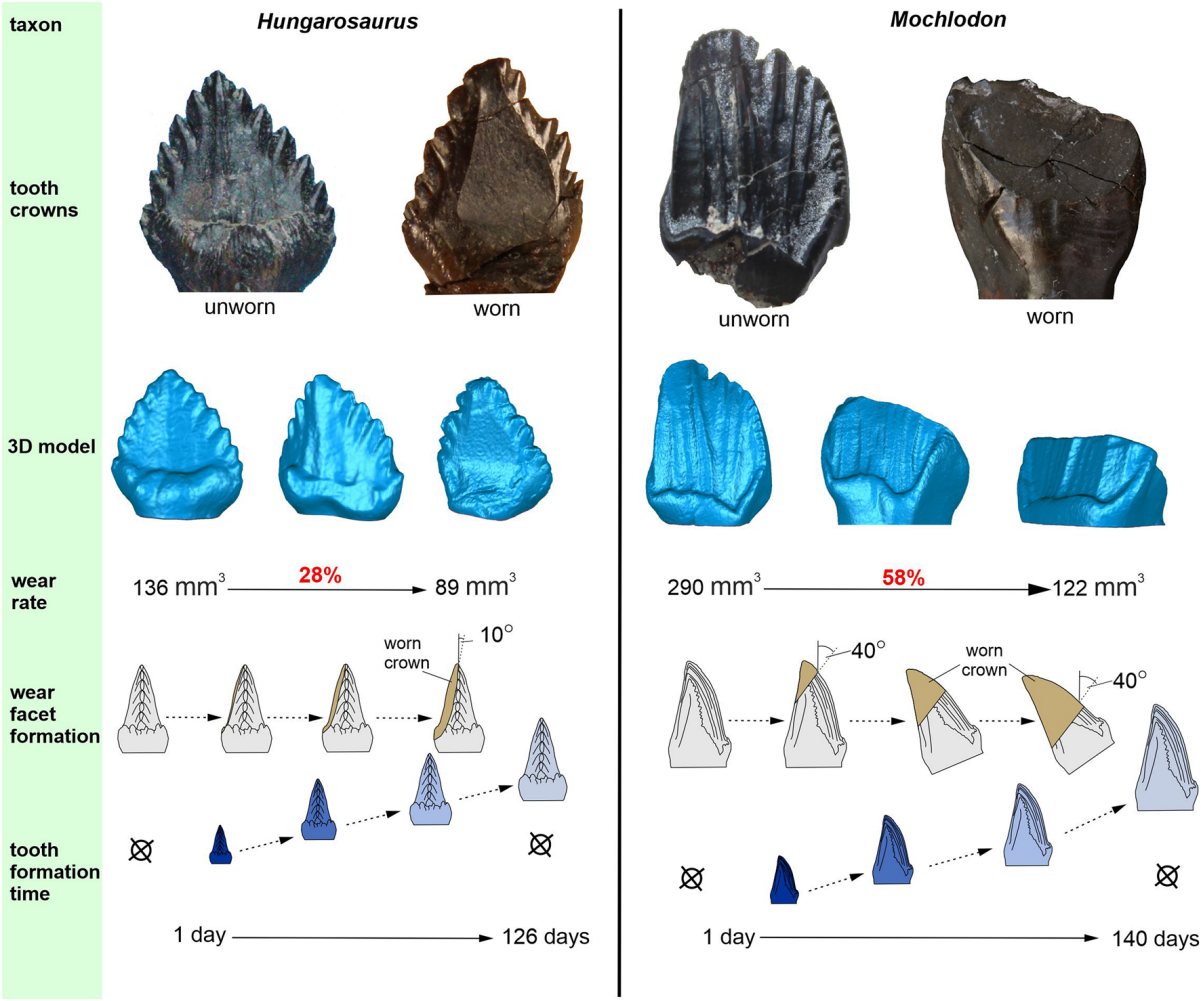
Comparisons between the stages of dental macrowear, wear rate, and tooth formation time in *Hungarosaurus tormai* and *Mochlodon vorosi*. Strongly worn crowns in *Hungarosaurus tormai* lost a maximum of 28% of the unworn crown volume, but this loss reaches 58% in *Mochlodon vorosi*. Note that inclinations of the wear facets differ between the two taxa, with much more steeply inclined facets in *Hungarosaurus tormai*. Also note that none of the taxa possess a replacement tooth beneath fully erupted but unworn teeth.

Some details of *Hungarosaurus tormai* tooth macrowear were described by^32^. In dentary teeth, the wear surface extends from the tip of the crown to the base of the cingulum and is bowl-shaped on the labial side of the crown (Figs. 2a, 3). This concave, bowl-shaped facet is most prominent on the basal third of the crown where the thickened cingulum is heavily worn (Fig. 3). A single facet is usually present but in some cases a double facet occurs, with these facets having slightly different orientations (^32^:Fig. 5e). Generally, the wear facet is situated predominantly on the central part of the labial surface, but in some instances it is shifted mesially or distally. Wear facets cover up to 60% of the labial crown surface (41.11 mm^2^/68.98 mm^2^ = 60% in MTM 2007.26.13 (173)) and are extremely steep, with an angle of 5–10° relative to the apicobasal axis of the crown. Wear facets on the upper teeth are less extensive (covering 20–50% of the lingual crown surface), and occur mainly on the apical region and are oriented at shallower angles (25–45°) relative to the apicobasal axis. Calculations of tooth crown volume based on the 3D models of the complete and strongly worn dentary teeth indicate that, on the most highly worn teeth, 28% of the complete crown volume was lost through wear (Fig. 3, and see “Discussion” section, below).

The macrowear features in *Mochlodon vorosi* were summarized by^38,60^. In the early stage of wear, the facet on the lingual side of the maxillary teeth is single, small (wear facet/occlusal crown area: 12.05 mm^2^/42.11 mm^2^ = 28.6% in MTM 2012.17.1_d) and steeply inclined (20–30° relative to the apicobasal axis). In later stages, the facet is more extensive, covering 60–95% of the crown surface (wear facet/occlusal crown area: 37.57 mm^2^/40.33 mm^2^ = 93% in MTM x41h; Fig. 3). The wear facets on strongly worn teeth are lower angled (45–65° relative to the apicobasal axis) than in *Hungarosaurus tormai*. However, this change in wear facet angle did not result in significant change to the angle of the occlusal plane, as the rotational eruption of the crown compensates for this (^8,38^, Fig. 3). Of the 19 worn maxillary teeth preserved only four bear double wear facets, where the difference in angle between the mesial and distal facets is only a few degrees. In the most heavily worn teeth these double facets merge into a single large facet.

Wear facets on the dentary teeth are usually steep (10–20° relative to the apicobasal axis of the crown) and, even in strongly worn crowns, this angle does not exceed 30–40°. Double wear facets are rare (or, at least, cannot be distinguished based on angle differentiation as clearly as in *Zalmoxes robustus*:^61^). However, the wear surface undulates slightly mesiodistally reflecting the alternation of some very shallow close-to-vertical grooves and ridges resulting from orthal jaw movement (see^38^). The volume of the tooth crown lost during wear is much greater in *Mochlodon vorosi* than in *Hungarosaurus tormai*. In the most heavily worn crowns, the wear facet reaches the cingulum and we calculate that up to 58% of the original complete crown volume was lost (Fig. 3, and see “Discussion” section below).

### Traditional and DMTA microwear analysis

**Figure 4:**
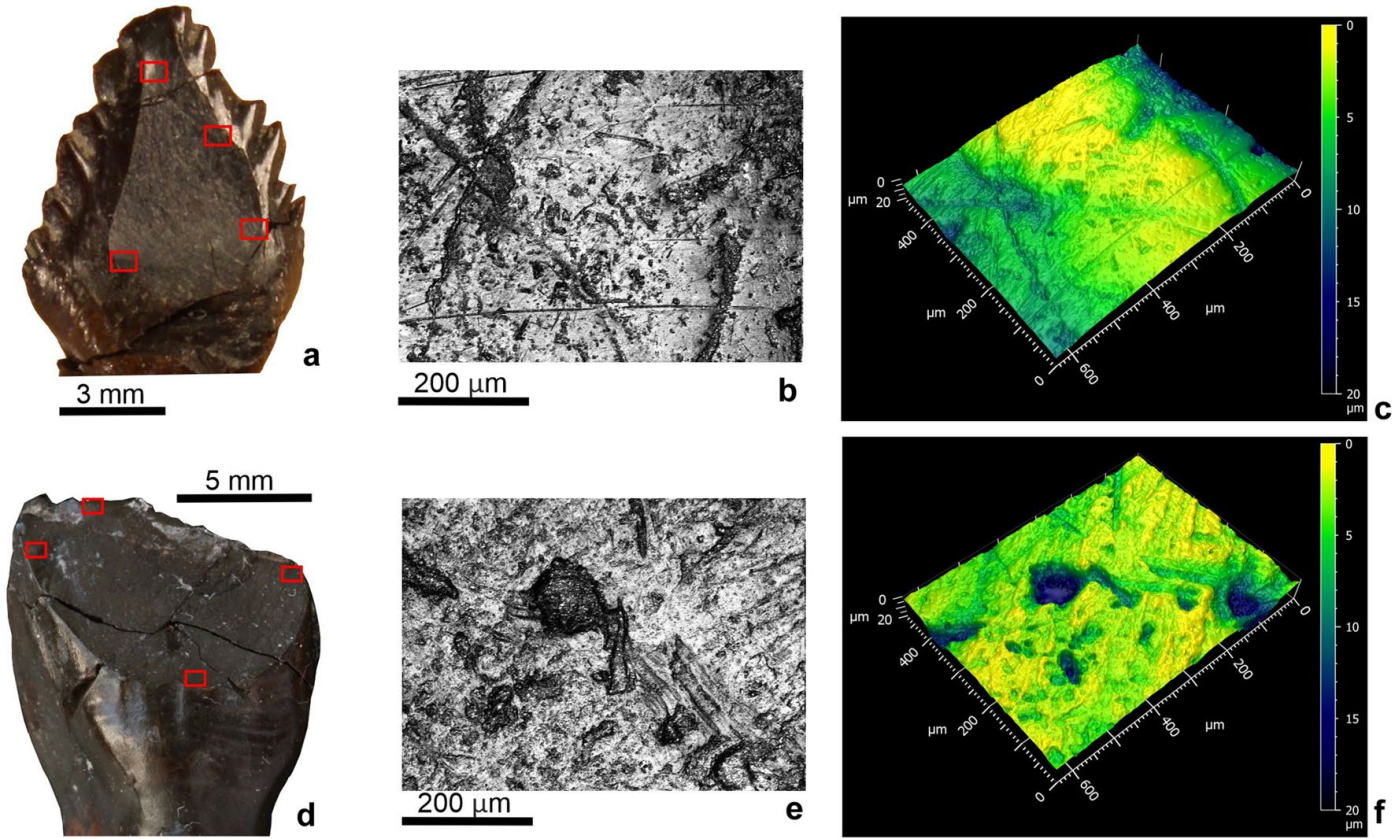
Microwear pattern details. (**a**) *Hungarosaurus tormai* tooth (MTM 2007.26.13) showing the position of the four areas imaged for traditional (2D) microwear and DMTA. (**b**) an example of one of the micrographs used for the traditional (2D) microwear analysis (PAL 2022.14.1.). (**c**) the same area in 3D for DMTA analysis. (**d**–**f**), showing the same for a maxillary tooth of *Mochlodon vorosi* (MTM 2012.17.1).

**Figure 5:**
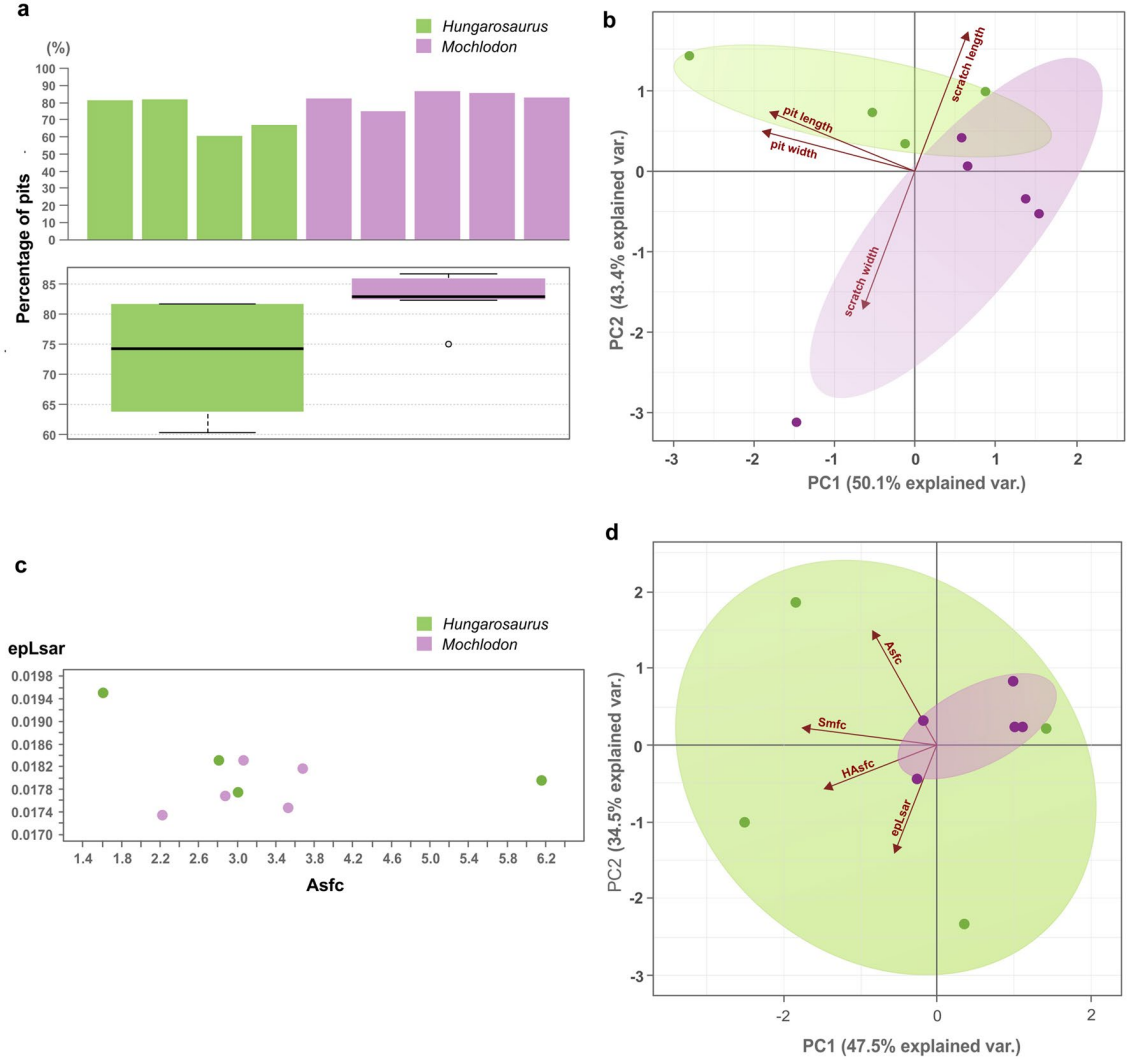
Results of the traditional (2D) microwear and DMTA. (**a**) Mean pit percentage based on four microgaphs taken from four teeth of *Hungarosaurus tormai* and five teeth of *Mochlodon vorosi*. (**b**) PCA analysis of traditional (2D) microwear data from *Hungarosaurus tormai* and *Mochlodon vorosi* using four microwear variables. Note the slightly larger pit dimensions in *Hungarosaurus tormai*. (**c**) Comparison of the DMTA data between *Hungarosaurus tormai* and *Mochlodon vorosi*. (**d**) PCA results of the DMTA analysis.

Four *Hungarosaurus tormai* and five *Mochlodon vorosi* teeth were analysed by traditional (2D) microwear and DMTA (3D) analysis in four study areas per tooth (Fig. 4). Conventional 2D analysis shows that both taxa have very high proportions of pits (~ 75% in *H. tormai* and ~ 83% in *M. vorosi*: Fig. 5a). When the 2D data are analysed using a PCA, PC1 explains 50.1% of the variance, while PC2 is responsible for 43.4% of the variance (Fig. 5b). PCA demonstrates that 2D microwear features of *H. tormai* and *M. vorosi* are similar, only the pits measured in *H. tormai* are slightly larger than those of *M. vorosi*. There is no significant difference in scratch lengths between the taxa, but scratches in *M. vorosi* are 12.1% wider.

The complexity (Asfc) and anisotropy (epLsar) values measured by the 3D analysis fall within similar ranges for both taxa (Fig. 5c). The anisotropy values are low and scattered over a very narrow range, whereas the complexity values cover a wide spectrum. In a PCA PC1 explains 47.5% of the variance, while PC2 is responsible for 34.5%. As in the biplot formed by ascf and epLsar, the PCA does not show any significant difference in the microwear textures of the two taxa (Fig. 5d). The two other variables, Smc and HAsfc (9 × 9) explain only very minor part of the PCA and are not relevant in the evaluation of the results.

### Tooth crown complexity

**Figure 6:**
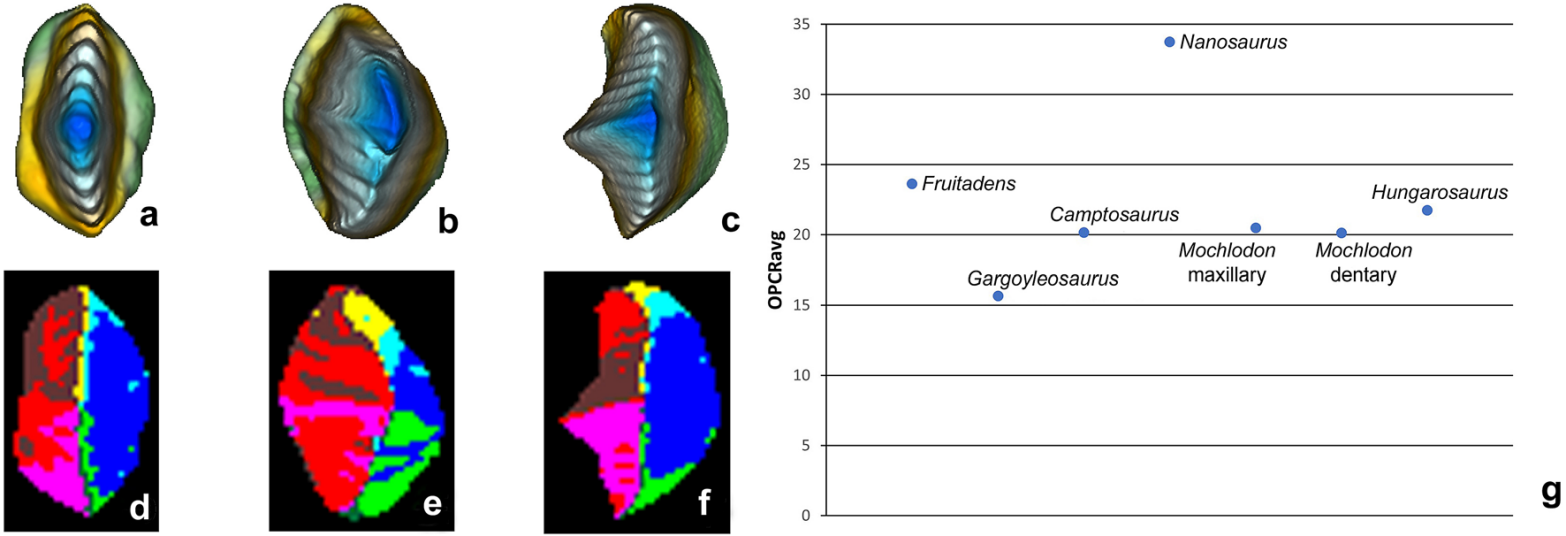
OPCR maps and average dental complexity of the unworn teeth of *Hungarosaurus tormai* and *Mochlodon vorosi* in relation to other ornithischians. Data for the latter taxa are taken from^20^; note that here data were calculated with the 3D OPCR method]. (**a**, **d**) *Hungarosaurus tormai*; (**b**, **e**) *Mochlodon vorosi* maxillary tooth; (**c**, **f**) *Mochlodon vorosi* dentary tooth. Note that OPCR values for *Hungarosaurus tormai* and *Mochlodon vorosi* are calculated only for one complete tooth. (**g**) Average dental complexity of the unworn teeth of *Hungarosaurus tormai* and *Mochlodon vorosi* in relation to other ornithischians.

OPCR analysis of an unworn, complete tooth crown of *Hungarosaurus tormai* revealed 21.75 patches with 50 RPT (nine with 25 RPT). This relatively high complexity value is related partly to the denticulate mesial and distal craniae, and to the crenulated cingula. While the maxillary and dentary teeth are morphologically identical in *H. tormai*, they differ slightly in *Mochlodon vorosi*. Nevertheless, tooth complexity values for the unworn maxillary and dentary teeth of *M. vorosi* are almost identical, at 20.5 and 20.175 patches per tooth with 50 RPT (8.25 and 9.125 with 25 RPT), respectively (Fig. 6). These values are very similar to those of *H. tormai* (see above) and fall in the middle range of modern saurians, in the insectivore and omnivore range^62^.

Compared with the complexity values measured in other dinosaurs^20^, the two Iharkút taxa, as expected, show similar patterns to those of other ornithischian dinosaurs (but see^20^ who used MorphoTester to calculate 3D-OPCR rather than Surfer Manipulator 2.5D-OPCR, which gives different absolute numbers, but similar patterns). They do not reach the highest value seen in *Fruitadens haagarorum* (36.5) or the average value (33.75) measured in *Nanosaurus agilis*, but are similar to that for the iguanodontian *Camptosaurus dispar* (20.15) (Fig. 6g). The teeth of *H. tormai* are more complex than those of the basal ankylosaur *Gargoyleosaurus parkpinorum* (15.68^20^), which might be related to the presence of a crenulated cingulum in the former.

## Discussion

### Tooth formation time, replacement rate and wear rate

Comparisons between the sympatric ornithischian dinosaurs *Hungarosaurus tormai* and *Mochlodon vorosi* reveal both differences and similarities in their dental characteristics. In spite of their markedly different functional tooth numbers and crown morphologies, the reconstructed maximum tooth formation time is quite similar (126 vs 140 days, see above). Tooth replacement was not rapid in either taxon, as the single generation of replacement teeth is not visible under newly erupted, unworn teeth but only becomes evident later, beneath teeth that are already worn. This suggests that new tooth mineralisation in both taxa started only after the functional teeth had begun to wear. However, the interval between the onset of wear and the appearance of a new tooth germ remains unknown. By contrast, the two taxa differ substantially histologically, and in *M. vorosi* the mean width of VEIB is almost twice that seen in *H. tormai*. Similarly, the tooth crown volumes in *M. vorosi* are 2–2.5 times greater than in *H. tormai*.

In *Mochlodon vorosi*, comparisons between a fully intact (volume [V]: 290.81 mm^3^) and heavily worn (V: 122.38 mm^3^)—but originally similarly-sized (based on mesiodistal crown length and labiolingual width)—maxillary tooth crowns show that up to 58% of the original tooth crown volume was lost as a result of feeding-related wear. In *Hungarosaurus tormai*, however, only 28% of the crown was lost, based on the volume difference between equally-sized intact (V: 136.28 mm^3^) and heavily worn (V: 98 mm^3^) teeth (see Supplementary data 1). *M. vorosi* has only 10 teeth per jaw quadrant, whereas *H. tormai* has 21. Theoretically, therefore, in *H. tormai* 798 mm^3^ (21 × 38 mm^3^) of the total original tooth volume in a single jaw quadrant was abraded in each tooth generation, compared to 1684.3 mm^3^ (10 × 168.43 mm^3^) for *M. vorosi*. Hence, in spite of similar tooth replacement rates and OPCR complexity in these taxa, *M. vorosi* wore through more than double the amount of tooth tissue lost in *H. tormai*, despite possessing much more thickly enamelled teeth (twice the enamel thickness). These differences in the extent of tooth wear, along with the markedly different crown morphologies of these taxa (compressed and phylliform in *H. tormai* vs more robust and chisel-like in *M. vorosi*), as well as the differences in orthal (*M. vorosi*) vs palinal (*H. tormai*) jaw movements during the powerstroke suggest that these taxa were exploiting the vegetation available to them in very different ways (see below).

Unfortunately, comparisons between the dental wear of *Hungarosaurus tormai* and other thyreophorans are currently limited to *Edmontonia* and *Pinacosaurus grangeri*^34,56^. *Edmontonia* exhibits strong wear on its teeth^30^, which differs from that present in *H. tormai* but is also due to occlusion; however, *P. grangeri* shows only minimal wear on the tips of its marginal denticles, suggesting the absence of occlusion and that the wear present was caused by tooth-food contact^33^. It is interesting to note that the different wear types and rates of wear seen among these three genera are not reflected in either tooth formation time or tooth replacement rate. *Hungarosaurus tormai* and *Edmontonia* had similar rates of wear with different mean widths of VEIB (18.7 µm and 13.9 μm, respectively), whereas *P. grangeri*, with minimal wear, had thicker widths of VEIB (15.7 μm) and a proportionally rapid tooth replacement relative to tooth size^34^. This suggests that the rate of feeding-induced wear in ankylosaurs (even in forms with occlusion) was not high enough to require the acquisition of rapid tooth formation or higher rates of tooth replacement (by increasing the number of replacement tooth generations), in contrast to the conditions seen in either sauropods^59^, hadrosaurids^56,63^, or ceratopsids^56,64^.

To our knowledge, tooth formation time has only been reported in one other thyreophoran, a taxonomically indeterminate stegosaur tooth from the Early Cretaceous of eastern Siberia^36^. This small tooth crown (height ~ 4 mm) possesses 95 VEIB with a mean width of 16.24 μm (measured on the basis of^36^. Skutschas *et al*.^36^ interpreted these observations as indicative of a short tooth formation time (95 days) and suggested a high tooth replacement rate. However, comparing these values with those of the above mentioned ankylosaurs, it seems that the Siberian stegosaur tooth was quite similar with respect to tooth formation. Its tooth size is almost identical to that of *Pinacosaurus grangeri*^34^ and the mean width of VEIB is also very similar (15.7 μm in the latter). Due to the more extensive, and perhaps faster, wear compared to that inferred in ankylosaurs^36^, a faster tooth replacement rate cannot be excluded, but further evidence from replacement teeth of this unnamed stegosaur taxon will be required to test this issue.

Although ornithopods were diverse, and their varied, often highly sophisticated feeding-related characters have been studied extensively (e.g.^3,7–9,21–23,37,63–65^), there are limited data on tooth replacement in non-hadrosaurid ornithopods, which consist primarily of descriptions of replacement teeth (see e.g.^58,66–68^). For non-hadrosaurids, tooth formation time and replacement rate has only been calculated in the rhabdodontids *Matheronodon provincialis*^57^ and *Mochlodon vorosi* (^38^, this study). These studies, along with the results presented here, further support the hypothesis of^58^ that the process and rate of tooth replacement in these relatively early-diverging ornithopods were quite similar to those of thyreophorans. In rhabdodontids and thyreophorans, the maximum number of replacement teeth per alveolus is one, but the number of replacement teeth in each jaw quadrant varies between groups. For example, while some non-iguanodontian and rhabdodontid ornithopods have replacement teeth in approximately every second alveolus, *Iguanodon bernissartensis* and *Owenodon hoggii*, among others, have them in almost every alveolus^69,70^ suggesting that tooth replacement rates increased in ankylopollexians.

As far as we are aware, the different lingual and labial mean tissue thicknesses of VEIB in *Mochlodon vorosi* have not been reported in any other ornithischian. Different tissue thicknesses between these features have been described in other archosaurs, but they were measured either in different orientations (e.g. labiolingual vs mesiodistal) or in different parts of the crown (e.g. at the level of the crown-root junction, cingulum or crown apex)^71^. This may result from the crown shape being narrower labiolingually than mesiodistally (e.g. in *Hungarosaurus tormai*). In the latter case, however, the difference in mean thickness of VEIB does not exceed 20–25%. The nearly double-thickness of VEIB in the labiolingual sections of *M. vorosi* teeth are always on the side of the crown involved in occlusion and where most of the resulting wear occurs (i.e. lingually on maxillary and labially on dentary teeth). It is hypothesised that the reason for this phenomenon is that the occluding side of the crown had to be mechanically more resistant, which required a greater amount of dentine to be formed on a daily basis on the working side of the crown than on the balancing (i.e. non- or less occluding) side, which is consistent with the thicker enamel also present on the working side of the tooth in the majority of cerapodan ornithischians.

In mammals^72^, noted that there are two ways to increase the durability of the dentition: 1) increase the wear resistance of the dental tissues; and/or 2) increase the amount of dental tissue available for wear. An example of the first solution is presented by hadrosaurs and ceratopsians, which convergently increased the complexity of their dental tissues^14,63,64^. The second solution can manifest either as increased tooth size, increased functional crown height (e.g. hypsodonty in mammals) or increased tooth number. An increase in tooth size has been documented in the rhabdodontid *Matheronodon provincialis*^57^ and the iguanodontian *Lanzhousaurus magnidens*^73,74^. An increase in relative crown height has been observed to some extent in sauropods^75^, while an increase in the number of teeth, in terms of accelerated tooth replacement, increased numbers of replacement teeth, and increased tooth counts are known in many dinosaur groups (e.g. sauropods^59,76^; hadrosaurids^3,63^; ceratopsids^14,77^). However, it appears that marked asymmetrical thickening of the dentine component of the tooth crown only occurs in some basal iguanodontians, where tooth replacement has not yet accelerated to the same high rates seen in later-branching taxa, but where continual tooth-tooth occlusion and/or less frequently replaced teeth with longer functional lives require more resistant teeth. Further studies are needed to confirm this hypothesis (Ősi et al. in prep).

### Wear pattern and possible dietary preferences

Traditional (2D) microwear analysis of *Hungarosaurus tormai* and *Mochlodon vorosi* shows pit dominance in both taxa (see above; Fig. 5a). Studies on herbivorous mammals have shown a general correspondence between a higher number of pits in browsers, whereas scratches are the dominant microwear features in grazers^78–82^. This variation can be attributed to a number of factors that have been also confirmed by experimental research, such as the shape, size, amount^83^ and hardness^84^ of exogenous particles, the amount of phytolite, or e.g. the water content of the food consumed^85^.

The small difference in the number of pits seen in the two taxa could be due to the fact that *Hungarosaurus tormai*, as a ground-level feeding ankylosaur, might have habitually consumed plants somewhat closer to ground-level than the bipedal *Mochlodon vorosi*. Nevertheless, the degree of pitting is similar in both taxa, which is consilient with their maximum vertical feeding ranges of up to ~ 1 m above ground-level (*H. tormai*, obligate quadruped, estimated body length of 4–4.5 m;^86^; *M. vorosi*, biped, estimated body length of 1.5–2 m^60^).

The low anisotropy and widely scattered complexity values obtained from DMTA for both taxa are consistent with the traditional 2D microwear results. Comparing the complexity values of the two dinosaurs with those of modern lepidosaurs^87^, we see that they are most similar to herbivorous, algivorous and frugivorous forms. However, for anisotropy, the ranges for herbivorous lizards are lower than those for the two dinosaurs studied. The DMTA ISO values measured in pterosaurs^88^ and sauropod dinosaurs^89^ are not directly comparable to those of the two ornithischian dinosaurs.

**Figure 7:**
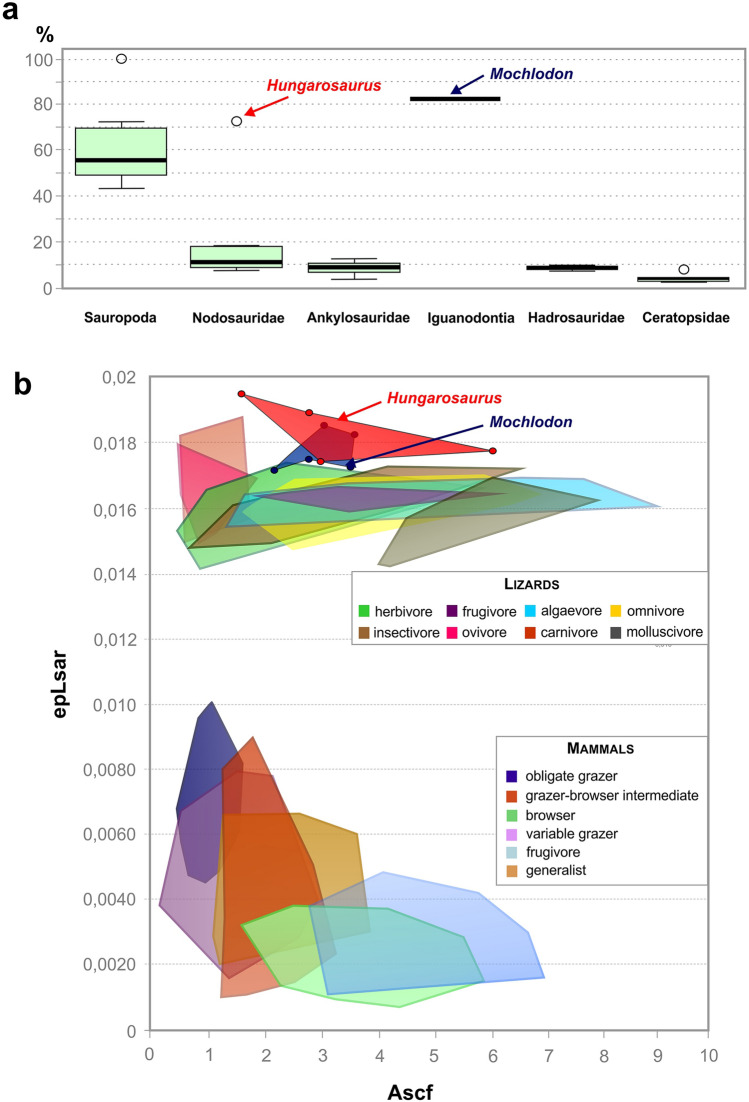
Microwear data comparisons. (**a**) Pit percentage (%) of *Hungarosaurus tormai* and *Mochlodon vorosi* compared to other dinosaur groups (data for other dinosaurs from^30^). Note the extremely high pit count ratios in the Hungarian taxa, which might result from a different data collection method (see “Methods”). (**b**) DMTA data for *Hungarosaurus tormai* and *Mochlodon vorosi* compared to those taken from extant lepidosaurs (data from^87^) and herbivorous bovid mammals (data from^52^).

A comparison of the DMTA values of the two dinosaurs with the tooth wear data of modern ungulate mammals shows that whereas they are most similar to browser and frugivore forms in terms of complexity, for anisotropy we see much higher values and no overlap with mammals (see Fig. 7b). Nevertheless, this observation has several caveats: the flora available to Cretaceous dinosaurs differed from that alive today; the dental tissues seen in the two clades are different (columnar vs prismatic enamel units in dinosaurs and mammals, respectively); in mammals DMTA was done on enamel, whereas in dinosaurs we could take our measurements only from the mantel dentine; and the micrograph sizes used in these studies also differed (our images taken with × 20 lens vs images taken with × 100 lens for the mammals in^52^). However, although the comparisons present in Fig. 7b cannot be interpreted uncritically, they do provide a useful baseline in the absence of other clear modern analogues.

Dental microwear studies have only been conducted on a few dinosaurs and focused primarily on basic characterisations of the wear features present (feature orientation, scratch-pit ratio, feature width) using 2D-micrographs (ankylosaurs^30,32,33^; ornithopods^7,8,25,30,90,91^; sauropods^75,90,92–94^; ceratopsians^19,30^). Mallon and Anderson^30^ reported much lower pit ratio counts in North American ankylosaurids and nodosaurids (pit ratios from 3.7 to 18.37%, Fig. 7a) compared to the high value (72.6%) in *Hungarosaurus tormai*. Whether this difference is due to different diets and/or differences in the microwear analysis method applied (see “Methods”, above) can only be determined by further research. Similarly, relatively high pit count ratio values were detected for sauropods by^94^ (Fig. 7a), most of which, however, clearly show a different, or at least greater range of browse heights than *H. tormai*. Interestingly, the highest pit count ratios occur in those sauropods where browsing height is close to ground-level (~ 0–1 m)^94^, which is identical to the browse height assumed for *H. tormai*. Whether, this similar high pit count could be indicative of a similar plant diet, despite the marked differences in feeding-related characters between the two groups (tooth morphology, tooth replacement, lack of precise occlusion in sauropods), requires further research.

For ornithopods, the numbers of microwear features present are known from only a few hadrosaurids^25,30,95^ and prior to this study were unknown in non-hadrosaurid iguanodontians. Williams *et al*.^25^ reported scratch dominancy and no pits in *Edmontosaurus*, whereas^95^ reported low numbers of pits in other North American hadrosaurids from different latitudes. Consistent with this,^30^ detected pit count ratios ranging from 4.67 to 18.64% in three different genera (*Prosaurolophus*, *Corythosaurus*, *Lambeosaurus*; Fig. 7a). The 82.5% pit count ratio in the rhabdodontid *Mochlodon vorosi* is much higher than the values documented in hadrosaurids. This difference is, however, consistent with the markedly different orientations of the wear facets, the much faster tooth replacement rates in hadrosaurids^3,63,64^, and their different chewing mechanisms (i.e. a shearing bite in rhabdodontids vs transverse or palinal grinding in hadrosaurids^7,8,25,26,96^). This suggests that, although there might have been some overlap in browsing height, *M. vorosi* and hadrosaurids consumed different food plants or plant parts. The high proportion of scratches in hadrosaurids suggests that, in contrast to *M. vorosi*, they consumed a diet obtained from near ground-level, similar to modern grazers (^25^, but see^95,97^). Conversely, *M. vorosi* appears to have consumed plants of taller stature, up to 1 m above ground-level, which might have either been tougher or processed more intensively, as also suggested for its close-relative *Matheronodon provincialis*^57^. Rather than increasing tooth replacement rates to compensate for wear, *M. vorosi* seems to have adopted a different strategy in which teeth were invested with extremely thick layers of dentine and a longer functional life (see above).

The many differences between the teeth of *Hungarosaurus tormai* and *Mochlodon vorosi* (see Table 2) suggest that the diets of these two sympatric herbivores differed. The labiolingually compressed, phyllodont, mesiodistally denticulate teeth, with a thin enamel layer, and steeply inclined wear facets on the dentary teeth, which lost less of their original volume to wear, suggests that *H. tormai* either consumed softer, easier-to-shear plants/plant organs than *M. vorosi* or processed its food far less intensively. In contrast to *M. vorosi*, which had a shearing orthal jaw action, *H. tormai* used a palinal powerstroke to partially masticate its fodder.

Based on its preserved stomach contents, the last meal of the early Albian nodosaurid *Borealopelta markmitchelli* included 84.9% fern fronds and 2.7% cycad leaves^98^. Fern and cycad fossils are known from the Iharkút site^41^, and based on their stature and physiognomy might have formed part of the diet for *Hungarosaurus tormai*. By the Santonian, however, flowering plants had become important understory plants in many ecosystems south of the circumpolar region^99,100^, so we can infer that this group might also have been an important dietary component for *H. tormai*.

### Possible causes of differential wear rates

The results presented above suggest that, in spite of similar microwear characteristics and tooth-replacement rates, the other significant differences in tooth wear between *Mochlodon vorosi* and *Hungarosaurus tormai* support niche partitioning between these sympatric taxa, as they likely exploited the available plant resources in different ways. In addition, the contrast between the greater amounts of tooth wear and adaptations for dealing with tough, abrasive foodstuffs in *M. vorosi* (see above) and the relatively lower degree of wear seen in *H. tormai* are not only interesting in terms of potential differences in diet, but also with respect to their potential metabolic demands. The estimated body mass (M_b_) of *M. vorosi* is ~ 41 kg^101^, while that of *H. tormai* is an order of magnitude greater at 650–688 kg^86,101^. When these parameters are considered alongside their different feeding adaptations, several more speculative avenues for further investigation emerge. Firstly, there is wide variation in the digestibility and calorific content of the different plant groups available during the Mesozoic (e.g.^102,103^), which might have led these taxa to select particular plant species or plant organs preferentially on the basis of their nutritional value. Secondly, due to its lower M_b_, *M. vorosi* should be expected to have higher mass-specific standard metabolic rates than the larger *H. tormai* (e.g.,^104^). By analogy with extant herbivorous mammals (e.g.^105^), this might suggest, in turn, that *M. vorosi* would have selected more easily digested or more nutritious fodder than *H. tormai* in order to fuel this increased demand. Thirdly, the extensive tooth wear and robust, thickened tooth crowns of *M. vorosi* are potentially indicative of greater dependency on oral processing than suggested by the less heavily worn teeth of *H. tormai* (perhaps supporting the need for more rapid assimilation in the former, as implied above), whereas *H. tormai* might have used a combination of oral processing and fermentative digestion, with the latter consistent with its greater M_b_, lower mass specific metabolic requirements (in concert with longer gut passage times) and greater overall food intake (facilitated by less time spent on oral processing). Finally, it is possible that the bipedal *M. vorosi* was more active than the quadrupedal and heavily-built *H. tormai*, which is potentially consistent with the need to release energy and nutrients from its food more rapidly.

## Conclusions

Using a broad range of morphological, histological, imaging and statistical methods, our comparative study of dentitions from the sympatric herbivorous dinosaurs *Hungarosaurus tormai* and *Mochlodon vorosi* reveals some similarities in their feeding relating characteristics but also highlights key differences indicative of niche partitioning, which might also indicate differences in dietary physiology. Compared to hadrosaurids and ceratopsids, both of these taxa can be characterized by relatively slow tooth replacement rates. The strongly asymmetrical deposition of dentine in *M. vorosi* tooth crowns appears to represent a novel solution to enable increased functional life for each tooth that has not been reported in any other ornithischian.

We provide new 2D microwear data and DMTA analyses to complement and expand upon the small number of existing studies on non-avian dinosaurs. The high pit count ratios, low anisotropy and scattered complexity observed in both *Hungarosaurus tormai* and *Mochlodon vorosi* strongly support browsing habits for both taxa, which would have consumed plants that were within the first metre above ground-level. However, the markedly different tooth morphology, tooth structure, patterns of tooth macrowear and jaw mechanics of these taxa suggest that they consumed different plant types or organs, providing a basis for ecological niche partitioning. These differences also seem to indicate that *M. vorosi* consumed tougher and/or more nutritious plant material and/or processed it more intensively than *H. tormai*. Moreover, when considered alongside the respective body masses of these taxa, we propose that the more extensive wear of *M. vorosi* might be consistent with a higher mass specific metabolic rate and/or increased activity levels. In addition, this study demonstrates the value of applying a wide range of different methods to dietary reconstruction provides stronger evidence for disentangling complex ecological signals, which would be difficult to detect if only a single proxy were used.

## Supplementary Information

Supplementary Information 1.

Supplementary Information 2.

## Data Availability

The datasets generated and/or analysed during the current study are available at https://doi.org/10.5281/zenodo.7313326.
